# Strategies for helping families prepare for birth: experiences from eastern central Uganda

**DOI:** 10.3402/gha.v8.23969

**Published:** 2015-03-31

**Authors:** Līga Timša, Gaetano Marrone, Elizabeth Ekirapa, Peter Waiswa

**Affiliations:** 1 Global Health, Department of Public Health Sciences, Karolinska Institutet, Stockholm, Sweden; 2 Department of Health Policy, Planning and Management, School of Public Health, Makerere University, Kampala, Uganda

**Keywords:** newborn health, maternal health, community health worker, birth preparedness, antenatal care, Uganda

## Abstract

**Background:**

Promotion of birth preparedness and raising awareness of potential complications is one of the main strategies to enhance the timely utilisation of skilled care at birth and overcome barriers to accessing care during emergencies.

**Objective:**

This study aimed to investigate factors associated with birth preparedness in three districts of eastern central Uganda.

**Design:**

This was a cross-sectional baseline study involving 2,010 women from Iganga [community health worker (CHW) strategy], Buyende (vouchers for transport and services), and Luuka (standard care) districts who had delivered within the past 12 months. ‘Birth prepared’ was defined as women who had taken all of the following three key actions at least 1 week prior to the delivery: 1) chosen where to deliver from; 2) saved money for transport and hospital costs; and 3) bought key birth materials (a clean instrument to cut the cord, a clean thread to tie the cord, cover sheet, and gloves). Logistical regression was performed to assess the association of various independent variables with birth preparedness.

**Results:**

Only about 25% of respondents took all three actions relating to preparing for childbirth, but discrete actions (e.g. financial savings and identification of place to deliver) were taken by 75% of respondents. Variables associated with being prepared for birth were: having four antenatal care (ANC) visits [adjusted odds ratio (OR_A_)=1.42; 95% confidence interval (CI) 1.10–1.83], attendance of ANC during the first (OR_A_=1.94; 95% CI 1.09–3.44) or second trimester (OR_A_=1.87; 95% CI 1.09–3.22), and counselling on danger signs during pregnancy or on place of referral (OR_A_=2.07; 95% CI 1.57–2.74). Other associated variables included being accompanied by one's husband to the place of delivery (OR_A_=1.47; 95% CI 1.15–1.89), higher socio-economic status (OR_A_=2.04; 95% CI 1.38–3.01), and having a regular income (OR_A_=1.83; 95% CI 1.20–2.79). Women from Luuka and Buyende were less likely to have taken three actions compared with women from Iganga (OR_A_=0.72; 95% CI 0.54–0.98 and OR_A_=0.37; 95% CI 0.27–0.51, respectively).

**Conclusions:**

Engaging CHWs and local structures during pregnancy may be an effective strategy in promoting birth preparedness. On the other hand, if not well designed, the use of vouchers could disempower families in their efforts to prepare for birth. Other effective strategies for promoting birth preparedness include early ANC attendance, attending ANC at least four times, and male involvement.

Maternal and child health and well-being have been prioritised in the 15 years through Millennium Development Goals 4 and 5 (1). Significant progress has been achieved globally in reducing mortality amongst mothers and children since 1990 (1), but more innovative strategies are needed to reduce bottlenecks to care-seeking, especially at the time of delivery (2). In Uganda, neonatal mortality remains high and has seen slower declines than maternal and under-5 mortality (3). The country has experienced varying rates of progress in improving the quality of care around the time of delivery, especially in rural areas where services are harder to access (1, 4). Even though most of the women attend antenatal care (ANC) during pregnancy, only 48% of mothers make at least four visits (1, 3). Despite an increase in the number of births assisted by skilled attendants since 1995 (1), the quality of care available may be low, with lack of equipment and skilled providers (3).

In the field of maternal health three basic delays have been identified as major bottlenecks in the provision and use of obstetric (5) and childcare services (6, 7): delays in deciding to seek care, reaching points of care, and receiving quality care at points of delivery. The promotion of birth preparedness and raising awareness of the possible complications and danger signs (DS) is one of the main strategies to overcome these delays and enhance the timely utilisation of skilled care, especially in low-resource settings (8, 9). Birth preparedness is defined as every pregnant woman and her family making a decision beforehand about the place of birth, service provider, and health facility, and having selected key items prior to delivery. It is generally thought that awareness of DS during the various stages of pregnancy and childbirth, and knowledge on when to seek care can reduce the first delay (5). Birth preparedness activities to overcome the second and third delays include planning where to give birth, identifying a birth attendant, planning for transportation, saving money, buying birth materials, and finding a blood donor (9). Each of these components is important and can be crucial in determining mothers’ and newborns’ survival. It is therefore essential to help pregnant women, their families, and the whole community to plan individually and together in order to ensure that no life is lost due to scarcity of birth preparedness items.

In Uganda, promotion of birth preparedness during counselling, and sensitising on DS are key interventions in the Minimum Health Care Package provided through ANC services since 2000 (10). However, it has been reported that counselling on birth preparedness is often not offered or, when offered, the service does not meet the national guidelines (11). In Uganda, only half (51%) of women are informed about pregnancy-related complications during ANC visits (3), with significantly lower coverage amongst rural women and those with less education (11).

To bridge access and demand creation gaps and delays, Uganda is scaling up a number of strategies, including use of community health workers (CHWs) or village health team (VHT) members (4, 10, 12, 13), and vouchers for transport or free services (14), among others. However, there is a dearth of evidence on how various supply and demand side strategies are associated with birth preparedness actions. The aim of this paper was to investigate strategies used to promote birth preparedness in order to increase access to maternal and newborn care. To achieve this, we assessed variables associated with birth preparedness in three districts of eastern central Uganda which use three different implementation strategies for promotion of safe births: use of CHWs (12), use of transport and service vouchers (14), and the routine government system which promotes birth preparedness counselling only at health facilities during ANC. The study was conducted as a baseline for a scale-up programme to improve maternal and newborn care learning from two successful pilot projects (12–15).

## Materials and methods

### Setting and study design

This study used a cross-sectional design in three districts (Buyende, Luuka, and Iganga) of the eastern central region of Uganda. The total fertility rate in this region is above the national average (3). Almost a quarter (24%) of 15–19-year-old females are pregnant or have a child, and 42% of women have an unmet need for family planning (3). During ANC visits just 32% of women are counselled about pregnancy-related DS; 67% deliver with the assistance of health professionals; and 29% have a postnatal check within 22 days of delivery (3).

The three districts were selected because they were close to each other, have people who speak the same language, and have similar cultural practices. However, Iganga has about 4% of its population classified as semi-urban as opposed to the other two that are completely rural (Table 1).

**Table 1:** Demographic and healthcare characteristics for the three study districts

Characteristics	Buyende	Luuka	Iganga
Residence	Rural, remote	Rural	Mostly rural
Population	248,000^a^	243,200^a^	466,200^a^
Health facilities (HF)	21	31	52
HF level III with MNC	4	6	6
HF level IV with MNC	1	1	1
Interventions	Voucher scheme	none	UNEST

MNC=maternal and newborn care.

a From health system assessment 2011 (16).

Buyende district had been home to the Safe Deliveries Study, which promoted access to care through use of vouchers (14). Pregnant women received vouchers for health services (to deliver in the nearest health facility, to attend ANC, and for postnatal care); and for transportation (to use local means, e.g. motorcycle, bicycle) to reach health facilities for ANC, delivery, and one postnatal care visit. The package promoted was comprehensive and included counselling on preparation for childbirth, but in practice was dominated by the vouchers. Provision of these vouchers removed access barriers and led to significantly increased use of maternal, newborn, and obstetric care services (14).

Iganga district was the setting of the Uganda Newborn Study (UNEST) which employed CHWs to promote maternal and newborn care through making home visits (12). CHWs conducted five home visits: two during the pregnancy, and three during the first week after delivery. During each pregnancy visit, CHWs counselled and showed women and families items they needed to have or steps to take as part of their birth preparations. In addition, they informed women about DS during pregnancy, and if present, the CHWs referred women to a health facility. On subsequent visits CHWs followed up how many and which actions had been taken to prepare for childbirth (12).

Luuka district had no additional intervention beyond the standard of care provided by the routine package of the Ministry of Health.

### Sampling and data collection

**Fig. 1:**
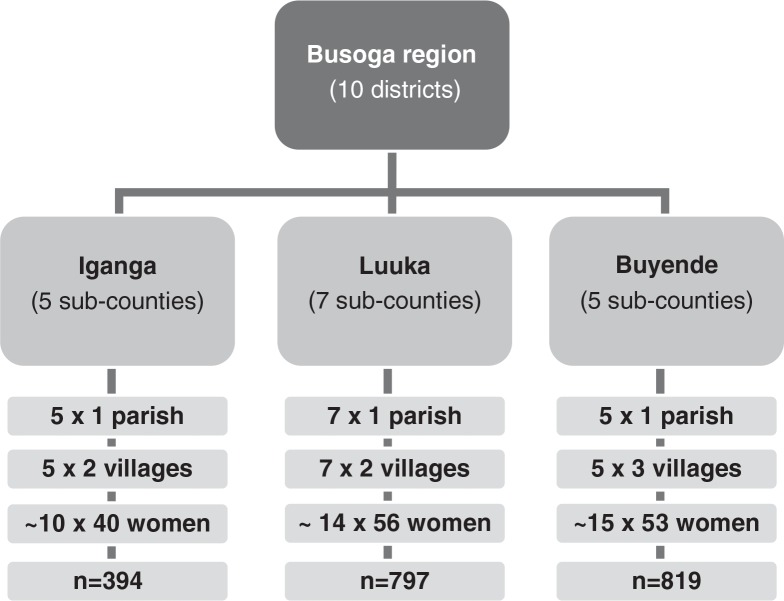
Sampling procedure in three study districts in eastern central Uganda.

Multistage sampling was performed by selecting parishes at the subcounty level and then villages within these parishes. In total, 17 parishes and 39 villages were included in the study (Fig. 1). Buyende had five subcounties and one parish was randomly selected. From each parish, subsequently three villages were chosen and about 53 mothers were interviewed from each village. Luuka had seven subcounties and seven parishes randomly chosen; in each parish two villages were selected for interviewing (about 56 mothers from each village). In Iganga, we randomly selected five parishes from five subcounties; and two villages per parish were picked (about 40 mothers from each village).

All women who had delivered a child in the past 12 months and were living in one of the selected villages were listed and selected. Exclusion criteria included a woman being ill at the time of the study, but we found none fitting that criterion. In total, 2,011 women were interviewed, but one woman was excluded from further analysis due to lack of data on outcome variables. Informed consent was obtained from the participants. The study was approved by the Makerere University School of Public Health Institutional Review Board and the Uganda National Council of Science and Technology.

A structured questionnaire with information on ANC, delivery and postnatal care, family planning, socio-demographic factors, and socio-economic data was used. The survey tool was translated into the local Lusoga language and was piloted and changed accordingly. The questionnaire was created following validated JHPIEGO guidelines (9). Thirty locally recruited field assistants with a minimum of secondary school education underwent a 3-day training (including 1 day of piloting) on data collection. Each interview lasted for about an hour. Data were collected from October 2011 to January 2012.

### Data analysis

Data were coded, entered, cleaned, and analysed using SPSS version 21.0 (17). A ‘birth prepared’ woman was defined as a woman who had taken three actions at least 1 week prior to the delivery: 1) chosen where to deliver; 2) saved money for transport and hospital costs; and 3) bought key birth materials (razor, thread, cover sheet, and gloves). These actions do not constitute the sum total of possible activities related to birth preparedness, but were chosen for analysis purposes and because they aligned with messages promoted in the three different settings during pregnancy.

If the respondent or her husband's main occupation was running a business or having salaried work, then she was coded as having ‘regular income’. On the other hand, if the main occupation of both respondent and husband was having a daily-wage job or being a farmer, then she was coded as having ‘irregular income’. Marital status was dichotomised within the categories ‘married’ and ‘not married’; widows, single, and divorced women belonged to the latter group. Education had three categories: ‘no formal education’, ‘primary’ which included finished or started primary schooling, and ‘secondary’ which contained secondary and higher schooling. The age groups of respondents were stratified as follows: under the age of 19 (less experienced women); 20–39 years old; older than 40 years (women who are experienced, but might be already in a risk group); and women who did not know their age.

The following were the independent variables: district, number of household members, number of children, sequence of pregnancy, marital status, mother's age group, religion, mother's education, husband's education, mother's income, husband's income, receiving information about DS, number of ANC visits, trimester during which the first ANC visit was undertaken, receiving care from a healthcare provider during the pregnancy, quintile of asset ownership, decision maker, and accompanied by husband to the place of delivery. For logistical reasons, no further verification of responses was done.

First, absolute and relative frequencies for all categorical dependent and independent variables were calculated. Second, the relationship between the outcome (birth preparedness) and selected independent variables was tested by Pearson's Chi-square test. Subsequently, all variables with *p* value<0.2 were inserted in a stepwise logistical regression model to investigate the association by obtaining the adjusted odds ratios (OR_A_) with 95% confidence intervals (CIs), thereby adjusting for confounders and effect modifiers likely to influence the outcomes. Absence of multi-colinearity between independent variables in the final model was tested and confirmed. Four different multivariable logistical regression models were tested: all cases together and one for each of the three study districts separately.

## Results

### Descriptive results

Most women in the study sample were married, more than half were Christian, and approximately one-third were Muslim (Table 2). The median age of the respondents was 25±6.6 years (ranging from 12 to 53 years). The proportion of husbands with at least secondary education was double the proportion of women with this level of education (39.1 vs. 20.2%). Most respondents and their husbands had irregular income, and 12.7% of women had no formal education.

**Table 2:** Socio-demographic characteristics of the sample

Characteristic	Frequency *n* (%)
District (*n*=2,010)	
Buyende	819 (40.7)
Luuka	797 (39.7)
Iganga	394 (19.6)
Marital status (*n*=2,007)	
Not married	179 (8.9)
Married	1,828 (91.1)
Respondent's age (*n*=2,006)	
≤ 19 years	326 (16.3)
20–39 years	1,507 (75.1)
≥ 40 years	69 (3.4)
Do not know	104 (5.2)
Religion (*n*=2,009)	
Christian	1,379 (68.6)
Muslim	612 (30.4)
Other	18 (0.9)
Respondent's education (*n*=2,008)	
No school	256 (12.7)
Primary	1,347 (67.1)
≥ Secondary	405 (20.2)
Respondent's income (*n*=2,006)	
Irregular	1,861 (92.8)
Regular	145 (7.2)
Husband's education (*n*=1,753)	
No school	108 (6.2)
Primary	959 (54.7)
≥ Secondary	686 (39.1)
Husband's income (*n*=1,753)	
Irregular	1,334 (76.1)
Regular	419 (23.9)

Median parity of the sample was 4±2.8 (range 1–15 children). Overall, 16.4% were primiparous mothers (Table 3). Median ANC attendance was 3±1.4 times (range 0–17 visits). Less than half of the women attended an ANC clinic at least four times, and just one-third of respondents went for their first session during the first trimester. One-fifth of pregnant women were told about the possible complications during the pregnancy period (2% of respondents were told about DS and 18% got information about DS and where to seek help). Very few respondents reported having been visited by a healthcare provider (doctor, nurse, CHW, or traditional birth attendant) during their pregnancy. In most families, the husband was reported as the decision maker regarding the woman's and newborn's health, and just 7.8% of women stated that they decided for themselves when to seek care and from where. Six of ten respondents reported they were accompanied by their husbands to the place of delivery (Table 3).

**Table 3:** Reproductive health and socio-economic patterns of the sample

Characteristics	Frequency *n* (%)
Number of children (*n*=2,009)	
1	329 (16.4)
2–3	597 (29.7)
4–5	434 (21.6)
≥6	649 (32.3)
ANC attendance (*n*=1,990)	
0–3 times	1,057 (53.1)
≥4 times	933 (46.9)
When was first ANC visit – trimester (*n*=1,967)	
1st	668 (34.0)
2nd	1,131 (57.5)
3rd	168 (8.5)
Told about pregnancy DS and where to seek help if they occur (*n*=2,005)	
None	1,595 (79.6)
Yes – DS	43 (2.1)
Yes – both	367 (18.3)
Visited by healthcare provider during pregnancy (*n*=2,010)	
No visits	1,935 (96.3)
Health professional	38 (1.9)
CHW/VHT	32 (1.6)
Other	5 (0.2)
Delivered at health facility (*n*=2,009)	
No	586 (29.2)
Yes	1,418 (70.8)
Decision making regarding mother's and newborn's health (*n*=2,008)	
Herself	157 (7.8)
Husband	1,674 (83.4)
Together	29 (1.4)
Other	148 (7.4)
Escorted by husband to place of delivery (*n*=1,887)	
No	739 (39.2)
Yes	1,148 (60.8)

CHW/VHT=community health worker/village health team.

### Birth preparedness across districts

**Fig. 2:**
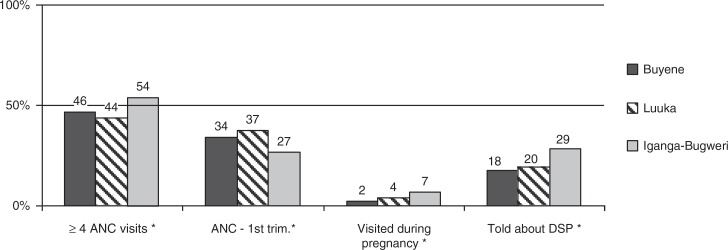
ANC practices among respondents (%) of three study districts. **DSP**=danger signs during pregnancy; *statistically significant differences (*p*<0.05).

**Fig. 3:**
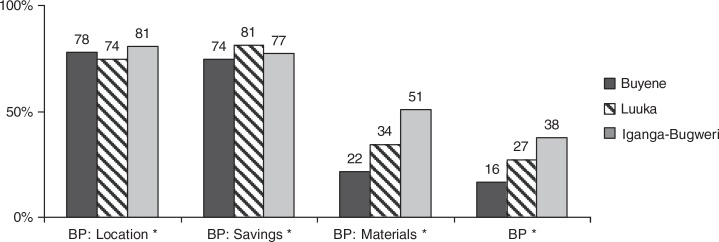
Respondents (%) taking individual and all three birth preparedness actions in the study districts. *Statistically significant differences (*p*<0.05).

As there were specific interventions carried out in the districts, a comparison was performed to evaluate distribution of different independent variables and birth preparedness in three study areas. Statistically significant differences observed across the districts include ANC attendance of at least four visits, first ANC session during the first trimester, a home visit during the pregnancy period, and receiving counselling on pregnancy-related DS, which were more common in Iganga than in the other districts (Fig. 2). Women from Iganga (CHW intervention) were more likely to take all three birth preparedness steps compared to women in Luuka (standard care) or Buyende (vouchers scheme) (Fig. 3). Although very few women performed all three preparation activities (Table 4), discrete birth preparedness actions were taken frequently. Fewer than 7% of women had not taken any actions to prepare for delivery.

**Table 4:** Birth preparedness^a^ characteristics

Characteristics	Frequency *n* (%)
Birth prepared^b^ (*n*=2,010)
Yes	497 (24.7)
No	1,513 (75.3)
Steps taken (*n*=2,010)
None	148 (7.4)
1 out of 3 steps	465 (23.1)
2 out of 3 steps	900 (44.8)
All 3 steps	497 (24.7)

a Birth preparedness steps: Identification of location for the delivery, saving for transport and other expenses, and procurement of four essential birth materials (razor, thread, sheet, and gloves).

b ‘Birth prepared’ was deemed a woman who had performed all three steps of birth preparedness by not later than 1 week before delivery.

Variables associated with birth preparedness in the logistical regression analyses varied among the three districts. The determinants of birth preparedness in Buyende district included attending ANC four times or more, counselling on DS and place of referral, contact with a healthcare professional during the pregnancy, coming from a household with highest asset ownership, and the respondent having a regular income (Table 5). However, if the husband was reported as the only decision maker regarding health issues, the odds of a woman being prepared for childbirth were reduced.

**Table 5:** Multivariable logistic regression analysis for associations with birth preparedness

	Pooled sample			Buyende			Luuka			Iganga		
			
	OR_A_	95% CI	*p*	OR_A_	95% CI	*p*	OR_A_	95% CI	*p*	OR_A_	95% CI	*p*
District
Buyende	**0.37**	0.27–0.51	0.0001									
Luuka	**0.72**	0.54–0.98	0.035									
Iganga	Ref											
ANC attendance
0–3 times	Ref			Ref			a			Ref		
≥ 4 times	**1.42**	1.10–1.83	0.007	**2.20**	1.38–3.51	0.001				**1.66**	1.05–2.61	0.030
When was 1st ANC visit – trimester
1st	**1.94**	1.09–3.44	0.024	a			**3.23**	1.22–8.60	0.019	a		
2nd	**1.87**	1.09–3.22	0.023				2.59	0.98–6.82	0.054			
3rd	Ref						Ref					
Told about pregnancy DS and where to seek help if they occur
None	Ref			Ref			Ref			a		
Yes – DS	0.49	0.20–1.20	0.119	0.00	0.00	0.999	0.41	0.12–1.42	0.158			
Yes – both	**2.07**	1.57–2.74	0.0001	**2.04**	1.21–3.44	0.007	**2.15**	1.41–3.28	0.0001			
Visited by healthcare provider during pregnancy
No visits	a			Ref			a			a		
Health professional				**14.40**	2.70–76.84	0.002						
CHW/VHT				0.00	0.00	0.999						
Other				0.00	0.00	1.000						
Decision making regarding mother's and newborn's health
Herself	a			Ref			b			a		
Husband				**0.29**	0.14–0.61	0.001						
Partners together				0.24	0.04–1.38	0.109						
Others				0.40	0.13–1.18	0.097						
Escorted by husband to the place of delivery
No	Ref			a			Ref			a		
Yes	**1.47**	1.15–1.89	0.003				**1.70**	1.16–2.50	0.007			
Quintiles of asset ownership index
1 (poorest)	Ref			Ref			a			a		
2	1.04	0.69–1.57	0.859	1.91	0.95–3.83	0.070						
3	1.12	0.74–1.69	0.589	1.43	0.67–3.06	0.361						
4	1.26	0.84–1.89	0.265	1.94	0.95–3.96	0.07						
5	**2.04**	1.38–3.01	0.0001	**3.69**	1.80–7.60	0.0001						
Respondent's income
Irregular	Ref			Ref			a			a		
Regular	**1.83**	1.20–2.79	0.005	**2.25**	1.09–4.64	0.029						
Husband's income
Irregular	a			a			a			Ref		
Regular										**2.04**	1.25–3.31	0.004

a Not significantly associated with the outcome

b OR and 95% CI with *p* value<0.05 are highlighted in bold.

In Luuka, variables that predicted birth preparedness were ANC visit during the first trimester, counselling on DS and place of referral, and husband escorting wife to the place of delivery. In Iganga, district birth preparedness was associated with at least four ANC sessions and the husband having a regular income.

When all cases were analysed together, we found the following to be independent variables associated with birth preparedness. Those coming from Iganga reported attending at least four ANC sessions, having an initial ANC visit during the first or the second trimester, being counselled on pregnancy-related DS and place of referral in case of complications, being accompanied by the husband to the place of delivery, coming from a household with highest asset ownership, and regular income.

## Discussion

Our findings show that the use of CHWs is an effective strategy in promoting birth preparedness. On the other hand, supply-side strategies such as use of vouchers to reduce delays in access to care and increase utilisation may hinder families from preparing for birth, if not well designed. A study in Western Uganda found that a low level of DS awareness among respondents combined with lack of preparation led to lower health-seeking behaviour, which could result in increased maternal and newborn mortality and morbidity (18). However, overall we found birth preparedness to be low in the three districts. Although discrete steps were taken by 75% of all women, the three actions under consideration were only carried out by 25% of respondents. In one study in Western Uganda, 91% of participants reportedly had saved money for delivery (18). This is high in comparison to other studies, where savings ranged from 36% in Ethiopia to 83% in Burkina Faso (19–23).The proportion of women taking all three identified actions was similar to another study in Western Uganda, which found 35% of respondents accomplishing at least three out of four actions (18).

We observed that three-fourth of the women in our sample population had financial savings, slightly more in Iganga and Luuka as compared to Buyende. The fact that areas with CHWs had better birth preparedness than areas with vouchers is interesting. It is suggestive that CHWs are able to change long-held cultural norms such as not preparing for the unborn child, probably because they are able to engage more on a one-to-one basis with individual women and with families on a more sustained basis, as compared to health workers in health facilities during ANC. Other findings also show the effectiveness of CHWs in terms of increasing referral compliance, and that training and support supervision can make them highly competent (13).

We identified that areas with vouchers had lower birth preparedness compared to those with CHWs. By nature the voucher scheme has one major aim, which is increasing utilisation of health facilities; it rarely includes empowerment of families to prepare for birth. Future voucher schemes need to be designed with empowerment of women, families, and communities as part of a comprehensive package; otherwise they can lead to dependency.

Besides use of CHWs, there were other effective strategies for promoting birth preparedness, including attending ANC at least four times, having an initial ANC visit during the first or the second trimester, being counselled on pregnancy-related DS, and male involvement. Obviously, there is a time limit for actions when ANC is sought later in pregnancy. However, only 34% of women across the three districts had their first ANC visit during the first trimester, and significant sociocultural barriers exist around revealing pregnancy early (11, 24). Studies in Ethiopia (22) and India (25) also found that ANC attendance of at least four times was associated with being prepared for birth.

Socio-economic status of the family was associated with being prepared for delivery in our study, that is, respondents or their husbands with regular income and those women from households in the highest asset ownership quintile. Education level did not contribute to birth preparedness in this study population, despite its importance elsewhere (18, 20, 25, 26). This could be due to the low levels of education generally across the population.

This study provides important information about the characteristics associated with care-seeking around the crucial time of childbirth in eastern Uganda. Two districts with and one district without any prior interventions were involved. However, there are several limitations to our study. All data collected relied on the respondents’ ability to recall their actions. To reduce recall bias only women who had delivered within the past 12 months were invited to participate. The same procedure was used in other studies in Western Uganda and elsewhere (26–28). Additional reporting bias could have occurred by the respondents giving answers perceived to be the best practice. The cross-sectional design of this study does not provide any causality inferences between the outcome and various independent variables, it only demonstrates associations.

## Conclusions

Engaging CHWs and other local structures may be an effective strategy in promoting birth preparedness. On the other hand, if not well designed, the use of vouchers could disempower families from making preparations for birth other than acquiring a voucher. Other effective strategies for promoting birth preparedness in this setting include early ANC attendance, attending ANC at least four times, and male involvement.
